# Delivery of Healthcare Resources Using Autonomous Ground Vehicle Convoy Systems: An Overview

**DOI:** 10.3389/frobt.2021.611978

**Published:** 2021-08-26

**Authors:** Calvin Cheung, Alireza Mohammadi, Samir Rawashdeh, Stanley Baek

**Affiliations:** ^1^ Electrical and Computer Engineering, University of Michigan, Dearborn, MI, United States; ^2^ Academy Center for UAS Research, Department of Electrical and Computer Engineering, United States Air Force Academy (USAF Academy), Colorado Springs, CO, United States

**Keywords:** convoy, platooning, medical transport, military doctrine, convoy performance metrics

## Abstract

Utilizing military convoys in humanitarian missions allows for increased overall performance of healthcare logistical operations. To properly gauge performance of autonomous ground convoy systems in military humanitarian operations, a proper framework for comparative performance metrics needs to be established. Past efforts in this domain have had heavy focus on narrow and specialized areas of convoy performance such as human factors, trust metrics, or string stability analysis. This article reviews available Army doctrine for manned convoy requirements toward healthcare missions and establishes a framework to compare performance of autonomous convoys, using metrics such as spacing error, separation distance, and string stability. After developing a framework of comparison for the convoy systems, this article compares the performance of two autonomous convoys with unique convoy control strategies to demonstrate the application and utility of the framework.

## Introduction

From a military perspective, a ground vehicle convoy is a column of two or more vehicles under a single leader, traveling from a set origin to an objective destination (Center for Army Lessons Learned, 2010). Military utilization of convoys has a long history, with doctrine on convoy utilization for the United States (U.S.) Army being described as early as 1847 in “An Elementary Treatise on Advanced-Guard, Out-Post, and Detachment Service of Troops” (Killblane, 2005). While the battlefield and vehicles have changed drastically throughout the years, the purpose of convoys has remained consistent: to control road movements to meet various logistical needs, such as movement of supplies, personnel, and equipment toward humanitarian and medical needs (Layer, 1993). Even though the topic of convoys has been thoroughly dissected and studied by the Army (Killblane, 2013; Killblane, 2015; MPRI Kuwait Observer Controller Team, 2003), the advent of autonomous vehicles has led to modernization efforts to improve convoys through the addition of autonomy. These efforts aim to improve convoy efficiency and performance, reduce the risks to the Soldier^1^, and decrease the overall cost of operations (Green, 2011).

In addition to military research, there are various other civilian organizations looking to develop and utilize autonomous ground convoy (AGC) systems. Efforts such as the Netherlands’ European Truck Platooning Challenge (European Truck Platooning, 2021) and the European Commission’s Safe Road Trains for the Environment Project (Waibel, 2011) demonstrated the interest of civilian governments in maturing autonomous convoy technology for improvements in safety, reduction in fuel consumption, and reduction of traffic congestion. In support of these efforts and commercial development, many companies, such as Peloton Technology (King, 2017), Scania (Francis, 2019), Daimler, Volvo, and Volkswagen (Vincent, 2016), are researching and developing autonomous convoy solutions.

At a high level, AGC systems have a lead vehicle and follower vehicles. Follower vehicles keep pace and formation with the lead per system requirements. This is normally done through the sharing of vehicle kinematics, intended maneuvers, or sensor data (cameras, GPS, LIDAR, wheel encoders, etc.) between the vehicles, which allows separate vehicles to actuate appropriately to meet the desired speed and formation (Nardini et al. 2018; Virdis et al., 2018; Campolo et al., 2018; Molinaro et al., 2018; Stea et al., 2018). The data are distributed wirelessly via a variety of different methods, such as vehicular ad hoc networks (VANET), Vehicle-to-Vehicle (V2V) communications, and Vehicle-to-Infrastructure (V2I) communications (Uysal et al., 2015; Jia et al., 2016; Lu et al., 2016; Wang et al., 2016; Zhang et al., 2016; Shen et al., 2016). Several different standards and protocols are used for network communications, such as dedicated short-range communications (DSRC) radios, 3G/4G Long Term Evolution (LTE) cellular networks, and roadside wireless sensor networks, to improve network coverage and throughput.

To properly gauge the performance of AGC systems, a proper framework for comparative performance metrics needs to be established. Past efforts in this domain have had heavy focus on narrow and specialized areas of convoy performance without considering the complex requirement of AGVs performing logistical operations, such as human factors, trust metrics (Davis et al., 2008), or string stability analysis (Feng, et al., 2019). We remark that developing human trust metrics about the performance of autonomous vehicles is a vibrant research area. In a recent work in Seet et al. (2020), Harvy et al. (2020), Bose et al. (2020), Dragomir et al. (2020), it is demonstrated that automation malfunctions such as deceleration failures do not deteriorate human trust by themselves. But rather the human driver’s inability for adaptive mitigation of the risk of negative outcomes such as risk of crashing resulting from those malfunctions adversely impacts the human trust. In Seet et al. (2020), Harvy et al. (2020), Bose et al. (2020), Dragomir et al. (2020), the human trust metric is reflected in changes in brain activity associated with action planning and motivational state. In addition, broad assumptions and simplifications were used in the analysis, such as the removal of lateral position considerations for convoy member vehicles, or the consistent existence in an information flow topology for robust inter-vehicle communications (Eben Li, et al., 2019). While the constraints, assumptions, and narrow focus areas of performance metrics that have been previously discussed are highly valuable for their intended purposes, a more generalizable approach is needed to compare performance across a larger swathe of autonomous convoy systems. The goal of this effort is to establish a framework for performance metrics of AGC systems for military humanitarian healthcare delivery missions by performing a review of AGC literature and comparing the findings to requirements found in Army doctrine relating to manned convoy systems. Based on these two critical pieces of information, we propose metrics for military AGCs. We will start with a brief historical exploration of the needs for autonomy in ground vehicle convoys and their utilization and benefits in humanitarian military delivery of healthcare. This will be followed by an exploration of overall manned convoy requirements. From there, we will survey the field of AGC efforts to determine common threads in the performance metrics to establish a generalized AGC performance metric framework to be used to compare the performance of future AGC efforts. Owing to military and commercial efforts having separate sets of needs and requirements, this article will focus on AGCs in the military domain, with an emphasis on humanitarian healthcare delivery, to be able to perform an in-depth look at the topic area.

The use of ground convoys to perform supply operations has been codified as an important part of an efficient strategy for the U.S. military as early as 1847 (Killblane, 2005). While modern military operations include more modern transportation systems such as rail lines, aircraft, and helicopters, ground vehicles still account for a significant portion of supply and equipment distribution. This reliance on ground vehicles was evident in Operation Iraqi Freedom, in which 98 percent of the military’s supplies and equipment were distributed by ground transportation (Green, 2011). In addition to general supply and equipment transport, humanitarian military missions and military healthcare support leverage convoys for delivery of healthcare personnel and resources to areas of conflict that nongovernment organizations are unable to safely assist. U.S. military medical units support remote regions of the world to provide medical support toward humanitarian aid (Gomez et al., 1996; Poropatich et al., 1996; Karinch et al., 1996; Zajtchuk et al., 1996). Depending on the location, humanitarian aid convoys can be leveraged to provide several tasks, such as protection *via* greater physical and psychological security when transporting healthcare supplies, escorting medical practitioners to areas of confrontation, and performing medical evacuations (Wolfson and Wright, 1995). Furthermore, medical aid may potentially be applied within the convoy vehicles themselves, depending on the extent of the need (Headquarters United States Army Reserve Command, 1997). Given the warfare scenarios and battlefields in which current military operations take place, the danger to the Warfighter and need for medical aid in convoys continue to grow (Harrell et al., 2007).

Vehicle platooning refers to linking of two or more vehicles in a convoy. In this article, we use the terms “platoon” and “convoy” interchangeably.

### Need for Military Autonomous Convoys

The unprecedented challenge of COVID-19 global pandemic requires an unprecedented global response. Given the military historical superiority in providing medical and humanitarian logistics, a very efficient way of distributing vaccine and pandemic-fighting supplies would be through military convoys. The global nature of the pandemic and the need for reaching out to the most remote areas necessitate the use of autonomous and robotic technologies. As an instance of medical logistics delivery, the autonomous military convoys can deliver screening devices for biosampling and image-guided diagnosis tools to an outbreak area. In particular, the autonomous convoys deployed to an outbreak area can install remote sensing devices (Di Lallo et al., 2021) such as the recently developed “Levelogger” machine^2^ for the rapid detection of the circulation of the COVID-19 virus within the impacted communities by sampling and testing wastewater in sewers and at wastewater treatment plants for the presence of the virus.

**FIGURE 1:**
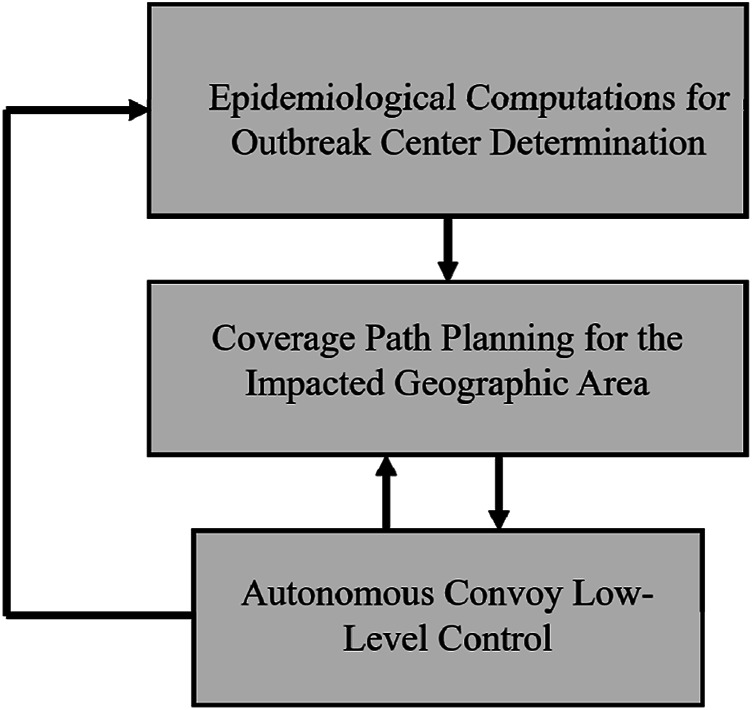
A hierarchical decision-making framework for deployment of military AGCs in combat against pandemics.

The coordination and command of such autonomous convoys need to be done in a proper hierarchical structure. At a higher level of hierarchy, there is a need for a supervisory control scheme that deploys the autonomous convoys to the areas that require mass vaccination and/or health service administration. For instance, the authors in David et al (2020), Baldassi et al (2020), Pio et al (2020) propose using Spatiotemporal Epidemiological Modeler (STEM) that can efficiently locate the centers of outbreaks and the course of epidemic trends. The obtained results on epidemic trends can then be used in coverage path planners (see, e.g., Nasirian et al. (2021)) that would ensure all the given points in an outbreak center are visited in a proper order. After the desired paths for an autonomous convoy in a geographic area are determined, low-level controllers will ensure that the trajectory tracking control objectives for the convoy are achieved (see Figure 1). This article is mainly concerned with achieving low-level control objectives in autonomous military convoys.

“Linear” and “Nonlinear” warfare scenarios are among the two main scenarios that can be defined for military autonomous convoys. In what follows, we argue that nonlinear warfare scenarios are more appropriate for medical logistics. In “linear warfare” scenarios, convoy operations are not prone to be attacked (Layer, 1993), where linear warfare is defined by conflicts in which opposing enemy forces generally proceeded forward. The geometry of a linear warfare battlefield implies that there is a “front” in which direct contact between forces is made, two “side” flanks that are often protected, and a secure “rear” area. Advancement in linear warfare means that forces at the front advance forward to clear and secure land. As the front moves forward, the noncombat assets in the rear progress as well, pushing forward and extending the secure rear (Harrell et al., 2007). Because of the aforementioned geometry, convoy operations are viewed as low risk in linear battlefields. Convoys are used to bring supplies and personnel from the rear to a forward position, traveling through secured areas that were far away from enemy combatants at the front battlefields. Many conflicts in U.S. history, including World War I, World War II, and Desert Storm, were linear warfare campaigns.

Despite the relative safety of convoy operations in linear battlefields, many modern conflicts and military operations in peacekeeping and humanitarian efforts are on a nonlinear battlefield. Indeed, in the epidemic spread models, computed using complex algorithms such as STEM (David et al., 2020; Baldassi et al., 2020; Pio et al., 2020), it can be observed that in contrast to linear battlefields, coverage areas do not have a defined front and secure rear area. Hence, there exists a need to use nonlinear battlefield techniques to deliver medical logistics to the outbreak area. The battlefield has a 360° area of operation with the center being a main operating base. In addition, many modern conflicts are against combatants that are using asymmetrical tactics. Asymmetrical tactics are strategies designed to harm a military’s assets without going up against the primary defenses and forces (Harrell et al., 2007). Prime examples of this are the targeting of unarmored convoys during supply operations with improvised explosive devices (IED), snipers, and sudden ambushes on stopped vehicles (Killblane, 2005; Layer, 1993). Past examples of U.S. military operations in nonlinear battlefields against combatants using asymmetrical tactics include the Vietnam War, humanitarian efforts on Bosnia and Somalia, and the conflicts in Iraq and Afghanistan throughout the first quarter of the 21st century (Layer, 1993; Harrell, et al., 2007). U.S. Department of Defense studies project most future conflicts will be on nonlinear battlefields against combatants using asymmetrical tactics, indicating a continued threat to the personnel and resources needed for convoy operations (Harrell et al., 2007). Given the threat of asymmetrical tactics on nonlinear battlefields, the U.S. Army is looking to leverage AGC systems for strategic and logistical benefits including reduction of danger to personnel and reduction in the costs of logistics.

Considering the recent COVID-19 events, the pandemic-induced supply chain requirements have been demonstrated to be different from traditional supply chains (see, e.g., Rutner et al. (2012), for the pre-pandemic status-quo views on commercial and logistical operations), where efficiencies are usually realized as cost savings. In particular, the main objective for distributing the COVID-19 vaccines (or in other future similar scenarios) has been to have a high throughput across the supply chain^3^. For this specific reason, the COVID-19-related logistical operations have been oriented toward humanitarian and wartime-like logistics, where highly responsive supply chains are constructed by the military in a way to meet the demand at any cost. With the surge of the COVID-19 variants across the globe, we believe that similar large-scale military supported logistical operations will be carried out to combat the pandemic. Indeed, the COVID-19 vaccine supply chain is set up in a completely different manner that is centered on the short-term measure of securing a sufficient supply of vaccines for the population spread across a vast geographic area.

The greatest threat to the safety of the personnel performing convoy operations in a nonlinear, asymmetrical battlefield are IEDs. IEDs are the main cause of battlefield casualties in Iraq and Afghanistan, accounting for 44% of the roughly 36,000 casualties from 2005 to 2009 (Green, 2011). By leveraging AGCs, vehicles can be operated with reduced direct human intervention, reducing the number of people needed in an operation, and thereby reducing the risk to human life by removing the personnel from dangerous situations. In addition to the lifesaving benefits, utilization of AGCs would provide tremendous cost savings as well. The cost of deploying a Soldier is estimated to be $2.1 M a year (Krumboltz, 2013), which means reduction of personnel needed has a built-in financial benefit. Furthermore, the use of autonomy in a convoy allows for greater precision in vehicle spacing due to the removal of human error, allowing for decreased spacing between vehicles. This decrease in spacing would reduce overall convoy length and provide fuel savings, which had been previously estimated to be between eight to twelve percent depending on the separation distance (Browand et al., 2004; McArthur et al., 2004; Radovich et al., 2004). Finally, from the humanitarian and healthcare mission perspective, increasing the autonomy of ground convoys reduces crew sizes, which is valuable in preventing the spread of infectious respiratory diseases, such as COVID-19. According to the Centers for Disease Control and Prevention, of the primary methods for protective against COVID-19 is to avoid close contact with others (Centers for Disease Control and Prevention, 2020). By leveraging greater levels of autonomy in convoys, the vectors of disease spread are decreased for both the warfighter and potential patients they may encounter as part of humanitarian military missions.

To reduce the threat to personnel, materiel, and medical supplies, and to reduce costs associated with convoy operations, the U.S. Army is looking to increase the utilization of autonomy in future operational concepts. Precision logistics, which entails the use of robotic autonomous delivery, is highlighted as a required Army capability set for sustained support of multi-domain operations (U.S. Army Training and Doctrine Command, 2018). In addition, the U.S. Army Robotic and Autonomous System Strategy specifically calls out autonomous convoys as a tool to enhance soldier survivability and reduce their exposure to hazardous situations (Maneuver Aviation and Soldier Division Army Capabilities Integration Center, 2017). With the high-level need being evident and understood by military leadership, a proper examination of the requirements is needed to be able to define metrics of success for an AGC system.

## Manned Convoy Requirements

Military doctrine outlines the fundamental set of principles that guides military forces in support of meeting its objectives (North Atlantic Treaty Organization Standardization Office, 2019). There exist four general types of military doctrine: Joint, Multinational, Multi-Service, and Service. While Joint, Multinational, and Multi-Service doctrine addresses processes common between multiple services (and nations, in the case of Multinational), every Service of the United States Armed Forces outlines Service specific doctrine defined to meet their idiosyncratic goals (U.S. Army Training and Doctrine Command, 2019). A thorough review of military doctrine can be performed to determine metrics and requirements of specific processes and systems for military needs. In this effort, we reviewed military doctrine to determine performance metrics for manned convoys. To limit the scope of the effort, we focused on service-specific doctrine from the U.S. Army due to their mission being most closely tied to the sustained utilization of ground convoys. In particular, our service-specific investigated topic is that of delivering medical logistics to the epidemic/pandemic outbreak areas.

All Army doctrine fits into a hierarchical structure with one of three classifications: Army Doctrine Publications (ADP), Field Manuals (FM), and Army Techniques Publications (ATP) (U.S. Army Training and Doctrine Command, 2019). Each of these publications serves a distinct purpose. ADPs contain the fundamental principles and foundations that guide Army actions in support of its objectives. FMs contain the tactics, procedures, and other relevant information in the execution of the principles described in the ADP. ATPs detail the flexible, non-prescriptive techniques to be used to perform Army missions, functions, and tasks. The doctrine has a hierarchy, with ADP on top, followed by FM, followed by ATP.

In addition to the doctrine, the Army also publishes training material, such as Training Circulars (TC) and Soldier Training Publications (STP). These documents can also contain information pertinent to the desired system performance and outcomes that are valuable in determining performance metrics. These documents, along with the aforementioned Army doctrine documents, are published from the Army Publishing Directorate (APD, 2021).

Table 1 lists the Army publications found to be relevant to convoy performance. An important characteristic of DoD publications is the Distribution Statement. Publications that have a Distribution Statement A label have been reviewed through the DoD Operational Security process and have been approved for public release (Office of the Under Secretary of Defense for Acquisition and Sustainment, 2012). Any other Distribution Statements, such as C or D, have restricted access and are not available to the general public. Owing to the limitation of availability in the information, the contents of those publications are not considered in this effort. However, they are included in Table 1 for the sake of completeness. The remaining Army publications that are approved for public release and pertinent to convoys are ATP 4-11 Army Motor Transport Operations; STP 55-88M14-SM-TG Soldier’s Manual and Training Guide MOS 88 M MOTOR TRANSPORT OPERATOR, SKILL LEVELS 1, 2, 3, and 4; and TC 21-305-20 Manual for the Wheeled Vehicle Operator. In the following sections, we will give a brief overview of the purpose of the publication, discuss its relationship to the convoy mission, and lay out the requirements that can be extracted toward the development of convoy performance metrics.

**TABLE 1:** Current Army publications relevant to convoy performance.

Publication number	Publication name	Distribution statement
ATP 4-01.45	MULTI-SERVICE TACTICS, TECHNIQUES, AND PROCEDURES FOR TACTICAL CONVOY OPERATIONS	Distribution D
ATP 4-11	ARMY MOTOR TRANSPORT OPERATIONS	Distribution A
STP 55-88M14-SM-TG	SOLDIER`S MANUAL AND TRAINER`S GUIDE MOS 88M, MOS 88 M MOTOR TRANSPORT OPERATOR, SKILL LEVELS 1, 2, 3, AND 4	Distribution A
TC 21-305-20	MANUAL FOR THE WHEELED VEHICLE OPERATOR	Distribution A
TC 4-11.46	CONVOY PROTECTION PLATFORM (CPP) COLLECTIVE LIVE FIRE EXERCISES	Distribution C

ATP 4-11 Army Motor Transport Operations details the Army’s doctrine in the utilization of motor transportation in the support of operations (United States Army Combined Arms Support Command, 2013). This support includes the movement of personnel, units, supplies, and equipment by vehicles. By performing these functions, motor transports allow for essential distribution capabilities, force sustainment, and extended operational reach, making them an integral part of the Army’s support and force sustainment. ATP 4-11 has information on the fundamentals, operations, and unit elements that make up motor transport operations. While the doctrine itself explicitly states that it does not go into details about convoy operations and battle drills, it still contains relevant information on how convoys are utilized, since they are used for motor transport. In the document, a convoy is defined as “a group of vehicles moving from the same origin to a common destination and organized under a single commander for the purpose of control.” This definition of a convoy is important to note, since the statement gives the following high-level requirement:

Requirement 1 - Two or more vehicles must be able to travel to a specified point.^4^

In addition, the various types of hauling required of motor transports specify the potential need for vehicles to make repeated trips, indicating the following requirement:

Requirement 2 - A convoy must have the ability to return to the original location after initially reaching the destination.

From the perspective of overall convoy system parameters, ATP 4-11 details multiple planning factors needed for convoy missions that shape the performance requirements of a convoy system. One important planning factor is the “rate of march.” The rate of march of a convoy mission is the average distance expected to be traveled by a given period of time. The need to be able to set a rate of march parameter indicates the following requirement:

Requirement 3 - Convoy system must have an adjustable rate of march.

**FIGURE 2:**
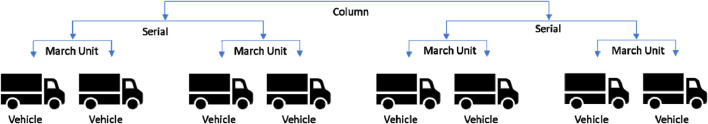
Convoy elements.

In addition to the rate of march, multiple planning factors related to convoy elements and associated gaps are discussed. A convoy can be broken down into smaller elements for organizational purposes. The smallest element is a march unit, which can have up to 25 vehicles. Next is a serial, which can consist of two to five march units. Following that is a column, which can consist of two or more serials. Figure 2 illustrates the breakdown of the described convoy elements. The proper gap spacing considerations of a convoy differ between vehicles and convoy elements. For vehicles, the gaps are defined by distance between vehicles, with the exact distance being set by a Convoy Commander. This indicates the following requirement:

Requirement 4 - Convoy system must have an adjustable gap distance^5^ between vehicles.

While convoy vehicles define the gap by distance, convoy element gaps are defined by a time gap. A time gap is the amount of time measured between convoy elements as they pass a specified point. Different convoy elements can have unique time gaps, such as march unit gaps and serial gaps, and are also set at the discretion of the Convoy Commander. This indicates the following requirement:

Requirement 5 - Convoy system must have an adjustable gap time between convoy elements.

Finally, ATP 4-11 also indicates that if a vehicle in a convoy is involved in a motor accident, then only the afflicted vehicle and its immediate successor should stop. All other vehicles in the convoy should continue the path when possible. This gives the following requirement:

Requirement 6 - Convoy systems must be able to complete its route even in the event of one or more vehicles leaving the system.

STP 55-88M14-SM-TG Soldier’s Manual and Trainer’s Guide MOS 88 M identifies the training requirements for Soldiers serving in the Military Occupational Specialty (MOS) of 88M, which is the designation for motor transport operators (U.S. Army Training and Doctrine Command, 2013). Rather than providing doctrinal guidance, STPs provide task summaries to help plan, conduct, and evaluate individual training in units. The task summaries provide information and instructions such as task conditions, task standards, performance steps, evaluation preparation, and performance measures^6^. Much of the information covers the processes necessary in performing motor transport, such as mission preparation, transportation of cargo, and motor pool management. In reviewing the task summaries, certain portions of the text were found to reinforce the need of the requirements identified in ATP 4-11. Specific training tasks indicated a need for a convoy to increase transit speeds in kill-zones, reinforcing the adjustable rate of march in Requirement 3. In addition, the need to situationally set gaps between convoy vehicles and elements depending on the desired convoy formation reinforced the Requirement 4 and Requirement 5. Aside from the reinforcement of previously described requirements, STP 55-88M14-SM-TG also identifies a new requirement based on the responsibilities attributed to the Convoy Commander relating to catch-up speed. Convoy Commanders are to set a catch-up speed that convoy followers must abide by. This indicates the following requirements:

Requirement 7 - Convoy system must be able to specify a maximum catch-up speed for individual convoy vehicles that fall behind.

The final convoy related publication available for public release is TC 21-305-20 Manual for the Wheeled Vehicle Operator. This TC describes operating practices, procedures, and techniques to efficiently operate a wheeled vehicle, including a chapter devoted to motor marches and convoys (U.S. Army Training and Doctrine Command, 2016). In this chapter, proper gap and vehicle speeds are discussed. The catch-up speed referenced in Requirement 7 is further enforced, and a speed-based gap distance is suggested as follows:

g=m∗s

with gap distance in yards (*g*), speedometer multiplier (*m*), and speed of the vehicle in miles per hour (*s*). The value of *m* is typically set at two but is variable as determined by the Convoy Commander. This adjustable gap calculation further emphasizes the need for Requirement 4 and Requirement 5.

In addition to the publications available for public release, Table 1 shows two additional documents: TC 4-11.46 Convoy Protection Platform (CPP) Collective Live Fire Exercises and ATP 4-01.45 Convoy Protection Platform (CPP) Collective Live Fire Exercises. TC 4-11.46 deals primarily with gunnery and training it handling threats (Frembling, 2012; Brooks, 2012), rather than topics pertaining to mobility performance requirements. ATP 4-01.45 “Multi-Service Tactics, Techniques, and Procedures for Tactical Convoy Operations,” contains tactics, techniques, and procedures relevant to leading of troops, employment of gun trucks, battle drills, and IED handling (ALSA, 2021). Both publications are restricted from public release to protect the information contained, and as such, are noted only for completeness.

### Challenges for Military Autonomous Convoys

In military healthcare delivery missions, there is a need for the autonomous convoy to be deployed to remote/rural areas, where the vehicles, which can belong to the class of Light Armored Vehicles (LAVs) or High Mobility Multi-purpose Wheeled Vehicles (HMMWVs), need to move on deformable terrains. Accurate and efficient tire models for deformable terrain operations are essential for performing low-level vehicle control (Taheri et al., 2015). As opposed to civilian truck convoys that often move on roads, a direct application of on-road tire models to simulate tire behavior on a deformable terrain such as soft soil is not viable. Therefore, the methods for performance evaluation and modeling of the wheeled vehicles on deformable terrains are affected by various terrain properties in addition to design and operational parameters. For instance, ruts that are formed into the ground by the travel of wheels and tracks can cause deterioration of vehicle mobility (Liu et al., 2009). Consequently, for each convoy member, there is a need for using advanced control schemes such as terramechanics-based path-tracking control laws that consider mismatched uncertainties due to interaction with soft soils (Taghavifar and Rakheja, 2019). In addition to issues arising from interaction with soft soil, the communication channels in-between the convoy members are subject to communication delay and packet losses (Pawar and Pan, 2016). This problem will become more pronounced if the convoy is being teleoperated from a remote base. Therefore, there is a need for delay prediction/compensation algorithms for control of these autonomous convoys in the field (Lu et al., 2018).

There exist numerous efforts in the development and improvement of AGC systems that focus on a number of different areas, such as control objectives, VANET factors, and control strategies (Jia et al., 2016; Lu et al., 2016; Wang et al., 2016; Zhang et al., 2016; Shen et al., 2016). Each effort defines customized measures of performance and success based on the research goals, but there is not a standardized set of high-level metrics to be used across AGC systems. Despite the lack of standardization, there is nonetheless commonality between how AGC efforts measure their system’s performance due to the common problem space that is being explored. The most common subjects that AGC efforts look to investigate are spacing policy and string stability; two closely related topic areas. By looking at metrics utilized in research efforts exploring these topics, we attempt to discern common threads in AGC metrics that can be used as a performance metrics framework for AGC systems for military utilization.

Spacing policy is the collection of methods, actions, and plans by which a convoy sets the desired distance between the vehicles (Rödönyi at al., 2014; Gáspár at al., 2014; Bokor at al., 2014; Palkovics at al., 2014). In general, the two most widely used platoon spacing policies are constant spacing and variable spacing. In a constant spacing policy, the separation distance between platoon members is independent of the speed of the controlled vehicle. The spacing error, ε_j_(t), of the *j*th vehicle, is as follows (Swaroop et al., 1994; Hedrick et al., 1994; Chien et al., 1994; Ioannou et al., 1994):

$${\text{ε}}_{\text{j}}\left(\text{t}\right)=x_{\text{j}-1}\left(t\right)-x_{\text{j}}\left(t\right)-L_{\text{j}}$$

where x_j_ is the *j*th vehicle’s position, x_j-1_ is the *j*th vehicle’s leader, and L_j_ is the following distance. In variable spacing, the spacing of the convoy vehicles is related to the vehicle’s speed, typically using a constant time headway approach. The spacing error δ_j_ is defined as follows (Ali et al., 2015; Garcia et al. 2015; Martinet et al. 2015):

$${\text{δ}}_{\text{j}}\left(t\right)=x_{j-1}\left(t\right)-x_{j}\left(t\right)-L_{j}-h_{w}v_{j}$$

where *h*_w_ is the time headway constant and *v*_j_ is the velocity of the vehicle j.

One of the primary goals of a convoy system is to reduce spacing error in accordance with the chosen spacing policy, which necessitates changes in control input to the follower vehicles. These changes and errors have the potential to propagate and amplify throughout the convoy, as each follower vehicle attempts to adjust their control parameters to reduce the error. A convoy system’s reaction to this propagation of error is referred to as “string stability,” with a convoy system being “string stable” if errors decrease, rather than increase, as they propagate through the convoy (Klančar et al., 2011). Intuitively, loss of string stability in a group of vehicles moving on a highway leads to undesired phenomenon such as the “accordion effect,” which leads to accidents and/or traffic jam.

More formally (Lu et al., 2017; Li et al., 2017; Huang et al., 2017):

$$\mathrm{||H}{(s)||}_{\infty }\leq 1$$

h(t)>0

where *h(t)* represents the ratio of spacing error between two consecutive vehicles and *H(s)* represents the Laplace transform of this function as follows.

$$h\left(t\right)=\frac{{\text{ε}}_{\text{j}}\left(t\right)}{{\text{ε}}_{\text{j}-1}\left(t\right)}\;$$

H(s)=L(h(t))

presuming constant spacing, with δ_j_(t) replacing ε_j_(t) for variable spacing.

**FIGURE 3:**
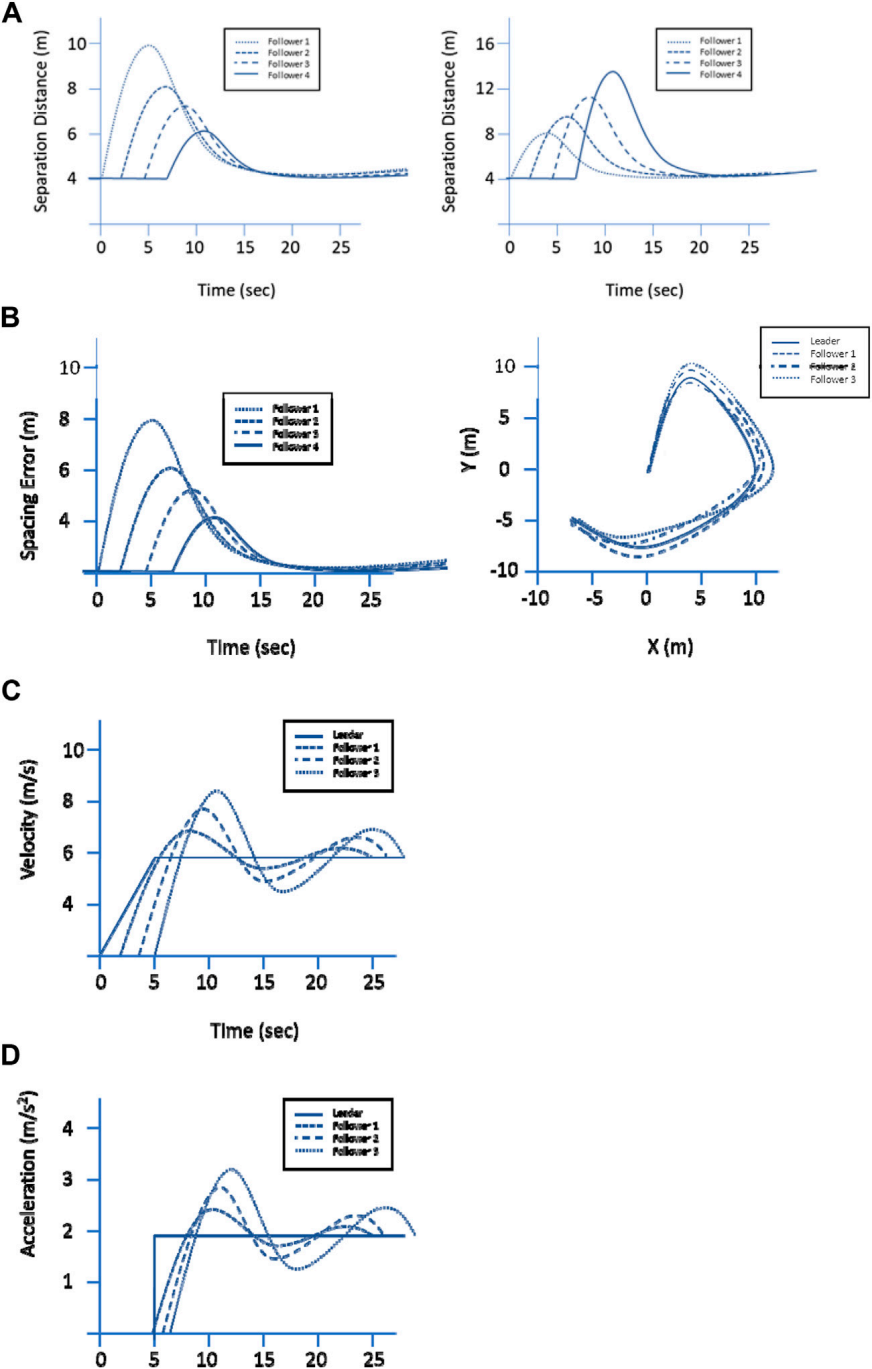
Prevalent metrics for Convoy Vehicle Performance for **(A)** separation distance, **(B)** spacing error, **(C)** vehicle velocity, and **(D)** vehicle acceleration.

It has been shown that string stability can be achieved in a convoy system with a variable spacing policy without any V2V communication, in contrast to constant spacing policy convoys that require some level of V2V communications to achieve stability (Guo and Yue, 2012). Experimental verification of string stability and adherence to spacing policy is often performed to validate that AGC systems are meeting the designed intent. The most prevalent metrics for experimentation can be split into separation distance, spacing error, velocity, and acceleration comparisons. Sample graphs for the various metrics can be seen in Figure 3.

When separation distance metrics are used, it presumes that the convoy vehicles start off with the desired spacing distance in a stopped state and looks at how the separation distance changes as the convoy system progresses throughout time. Given that the separation distance is not static in a convoy system using a variable spacing policy, this metric is normally used when examining convoy controllers using a constant spacing policy (Guo and Yue, 2012; Ali et al., 2015; Garcia and Martinet, 2015; Lu et al., 2017; Li and Huang, 2017). Figure 3A shows a representative separation distance graph, with a string stable system on the left, and a string unstable system on the right.

Another metric that is often used in gauging autonomous convoy performance is spacing error over time. Convoy systems that leverage variable spacing tend to use spacing errors as the experimental metric, given the variable nature of separation distance between the member vehicles. Convoy systems that are string stable will show spacing errors that decrease along the follower vehicles (Li and He, 2018; Rödönyi et al., 2014; Gáspár et al., 2014; Bokor and Palkovics, 2014; Liu et al., 2014; Gao et al., 2014; Xu et al., 2014; Liu and Cheng, 2014; Gong et al., 2016; Shen and Du, 2016), as shown on the left side of Figure 3B. A separate way that spacing error is commonly used is in comparing convoy member vehicle offset to the desired trajectory, which is known as path following error (Klančar et al., 2011; Zhao et al., 2017; Goi et al., 2010; Klančar et al., 2009), as shown on the right side of Figure 3B. The path offset is then used to calculate an error metric, such as with a root mean squared error approach. This is typically used when systems are looking to examine path replication, rather than string stability.

An additional common metric that was discovered in gauging autonomous convoy performance was vehicle velocity. Given that the primary goal of a convoy in motion is to have followers maintain a certain gap distance with a lead vehicle, followers will always be aiming to converge to a velocity that matches its leader (Lu et al., 2017; Li and Huang, 2017). As such, it is important to note if a convoy’s ability to have the vehicles reach a desired velocity is string stable. Since disturbances that are exerted on an individual convoy member can adversely deteriorate the string stability of the whole convoy, ensuring disturbances to vehicle velocity are not amplified throughout a convoy’s followers, and noting time to convergence at the desired velocity, are important factors when examining performance (Guo and Yue, 2012; Liu et al. 2014; Gao et al. 2014; Xu et al. 2014; Liu and Cheng, 2014; Gong et al. 2016; Shen and Du, 2016; Lu et al., 2017; Li and Huang, 2017; Li and He, 2018). Figure 3C shows a representative velocity graph for a system that is not string stable in terms of velocity.

The final metric we reviewed in gauging autonomous convoy performance is control effort acceleration for the vehicles. If the control effort is not string stable, the reliability of vehicle operation can be put into jeopardy, as amplification of acceleration requests can exceed the limits of the vehicle’s capabilities (Öncü, 2013). As such, string stability for acceleration is important for not only convoy performance, but overall safety and maintenance of the vehicles. Indeed, since the acceleration is proportional to exerted forces, acceleration string stability metric can be directly used to study the effect of exerted disturbances on the convoy dynamics and its position/velocity string stability metrics. Ensuring disturbances to control effort are not amplified throughout a convoy’s followers, and noting time to convergence at the desired acceleration, are important factors when examining performance (Guo and Yue, 2012; Liu et al., 2014; Gao et al., 2014; Xu et al., 2014; Liu and Cheng, 2014; Rödönyi et al., 2014; Gáspár et al., 2014; Bokor and Palkovics, 2014; Gong et al., 2016, Shen and Du, 2016; Li and He, 2018). Figure 3D shows a representative acceleration graph for a system that is not string stable in terms of acceleration.

## Framework for Comparing Autonomous Convoys for Military Use

Through analyzing Army doctrine, we have derived generalized requirements that can be leveraged to apply to autonomous ground vehicle convoys in assessing their ability to perform Army missions. By leveraging common autonomous convoy performance metrics to gauge how well the requirements are being met, we can develop a framework that can be used to assess AGC performance across different systems. For greater clarity, we will be classifying the manned convoy requirements into three categories of analysis: Goal Specification, Spacing Policy, and System Parameters. Refer to Table 2 for the specific categorization of requirements. The following sections will examine which metrics are best utilized for comparison for each different category of requirements. An example application of the framework will then be shown by examining a simulated AGC.

**TABLE 2:** Categorized manned convoy requirements.

Category	Requirement
Goal Specification	Requirement 1—Two or more vehicles must be able to travel to a specified point
	Requirement 2—A convoy must have the ability to return to the original location after initially reaching the destination
	Requirement 6—Convoy systems must be able to complete its route even in the event of one or more vehicles leaving the system
Spacing Policy	Requirement 4—Convoy system must have an adjustable gap distance between vehicles
	Requirement 5—Convoy system must have an adjustable gap time between convoy elements
System Parameters	Requirement 3—Convoy system must have an adjustable rate of march
	Requirement 7—Convoy system must be able to specify a maximum catch-up speed for individual convoy vehicles that fall behind

### Goal Specification

Per Army doctrine, a convoy system must be able to travel to a designated point (Requirement 1) and optionally return to the original point of departure (Requirement 2) as dictated by mission needs. This indicates that there is a desired path and goal that the AGC is meant to follow as closely as possible, and deviation from said path is undesirable. Given these needs, offset spacing error, as shown on the right in Figures 3B, is the most appropriate metric to compare the performance of AGC systems. The desired position of lead vehicles and the relative position of the follower vehicles can be used to calculate the offset between the desired and actual positions. This metric can be used for both Requirement 1 and Requirement 2, since Requirement 2 can be considered an extension of Requirement 1 with multiple goal points. To evaluate overall offset spacing error performance, we will adapt evaluation metrics for position tracking from the domain of computer vision (Needham and Boyle, 2003) due to the similar goals between leader following and position tracking.

Another aspect of goal specification is that convoy systems must be able to complete their route even if one or more vehicles leave the convoy (Requirement 6). Once again, this looks at how well an AGC follows the path of a lead vehicle, with the added complexity of having a convoy follower needing to modify which vehicle it is following to ensure that a disabled follower vehicle does not cause all followers to halt. This also can be examined by leveraging spacing offset error, as shown in Figure 3B, as a metric of comparison. Vehicles that are unable to continue with the convoy will produce a greater overall error in the system, giving a data point to compare between different AGC implementations.

### Spacing Policy

As previously defined, spacing policy is the collection of methods, actions, and plans by which a convoy sets the desired distance between the vehicles (Rödönyi et al., 2014; Gáspár et al., 2014; Bokor and Palkovics, 2014). The two primary categories of spacing policies are constant spacing and variable spacing. The doctrinal requirements align with the two categories of spacing policy, with the need for an adjustable gap distance (Requirement 4) aligning with constant spacing, and the need for adjustable gap time (Requirement 5) aligning with variable spacing. The key areas of comparison for spacing policy performance are string stability and time to convergence. A string stable system will not propagate errors throughout a convoy, meaning that convoy followers will more closely adhere to the desired speed and position. In addition, string stability allows the overall convoy to reach its desired end state more rapidly, meaning that the time needed for each follower vehicle to converge to the desired system parameter is lower. Therefore, string stability-related metrics, such as amplification of the response of follower vehicles and overall time it takes for the follower vehicles to converge to the steady state (Guo and Yue, 2012; Liu et al., 2014; Gao., 2014; Xu., 2014; Liu and Cheng, 2014; Rödönyi et al., 2014; Gáspár et al., 2014; Bokor and Palkovics, 2014; Gong et al., 2016; Shen and Du, 2016; Li and He, 2018), are appropriate tools for comparison. Examples can be seen in Figure 3. The choice of which area to examine for string stability (spacing, velocity, acceleration, etc.) is dependent on the mission goals that the AGC is attempting to meet.

### System Parameters

The requirements categorized under System Parameters deal with overall convoy system settings. Army doctrine defines the need for an adjustable rate of march (Requirement 3) and an ability to set a maximum catch-up speed for the convoy follower vehicles (Requirement 7). Overall, they impose qualifiers and restrictions to how the AGVC meets spacing policy requirements. These requirements can be analyzed with a binary success or failure by monitoring the overall convoy velocity and the speed of the individual vehicles. If comparisons of greater granularity are needed, the distinction can be made by examining the string stability-related metrics described in Spacing Policy at different rates of march and catch-up speeds. This would entail examining multiple runs of a convoy system and changing the system parameter to be evaluated between each run. The results of each run can be examined for string stability-related metrics, such as amplification of the response of follower vehicles and overall time it takes for the follower vehicles to converge to the steady state. The examples can be seen in Figure 3. Changes in these measurements between the various runs can then be noted for an AGC system, which could then be compared with how changes affected performance for other AGC systems.

## Application of Framework

To apply our framework for comparing autonomous convoys, we leveraged the Autonomous Navigation Virtual Environment Laboratory (ANVEL), “an interactive, real-time engineering modeling and simulation (M&S) software tool built specifically to assist in the research, design, testing, and evaluation of intelligent ground vehicles (Quantum Signal, 2018).” ANVEL features Python application programmer interfaces to set up and control autonomous convoys in an M&S environment.

Two different convoy following controllers were used in our application of the framework. One convoy controller utilized a Pure Pursuit method for geometric path tracking, in which the center of the rear axle is used as the reference point on the vehicle to compute a steering angle toward a look-ahead point at a fixed distance (Amidi and Thorpe, 1991). The other convoy controller utilized the Stanley method for geometric path tracking, in which the front axle is used as the reference point, and both the heading error and cross-track error are used to find the proper steering angle (Thrun et al., 2006). These two control schemes represent the two ends of the spectrum of geometric/kinematic controllers in terms of dependency on the number of to-be-tuned parameters where the Pure Pursuit controller relies less on the system parameters while the Stanley controller, which was the winner of DARPA challenge 2005 (Buehler et al., 2007) relies on more tunable parameters.

The following schematic diagrams depict the schematic diagrams associated with the Stanley and pure pursuit control schemes. Some remarks are in order (see, e.g. Snider, 2009, for more detailed explanations). Both pure pursuit and Stanley controllers belong to the family of path tracking algorithms, namely, algorithms that make a vehicle to execute a globally defined geometric path by applying appropriate steering commands that guide the vehicle along the path. The goal of any path tracking algorithm is to simultaneously minimize the lateral distance between the vehicle and the defined path, to constrain the steering control inputs to smooth input commands, and to minimize the heading of the vehicle and the defined path heading.

**FIGURE 4:**
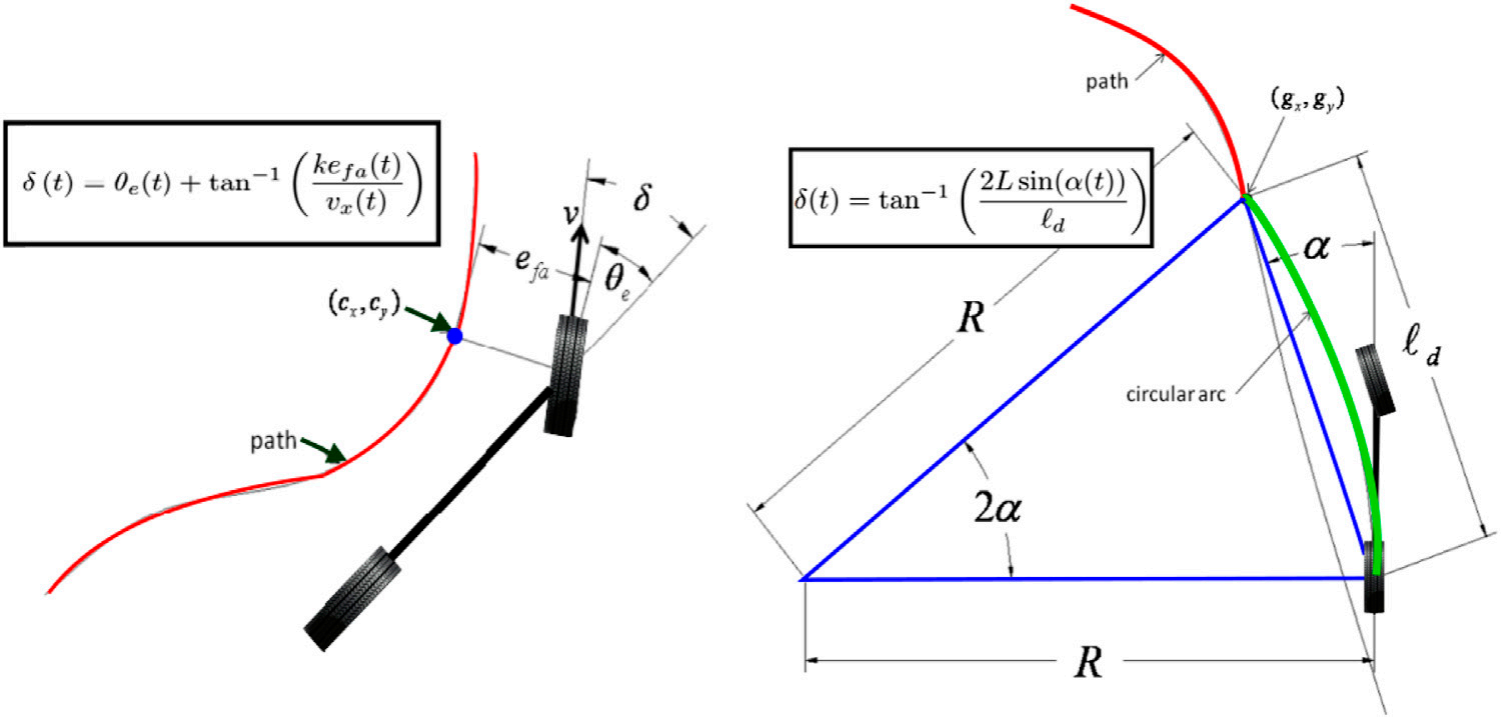
Stanley (left) and pure pursuit (right) controller schematic diagrams (recreated from the schematic diagrams in Sinder, 2009).

As it is demonstrated in Snider (2009), pure pursuit controllers are essentially proportional controllers with a proportional gain of $2/l_{d}^{2}\;$ acting on the steering angle dynamics. Indeed, if the curvature of the circular arc in Figure 4 (right) is given by κ, then it can be shown that $\kappa =\left(2/{\text{l}}_{\text{d}}^{2}\right){\text{e}}_{{\text{l}}_{\text{d}}},$ where $e_{l_{d}}:=l_{d}\sin\left(\text{α}\right)$ is the cross-track error. A geometric interpretation of the parameter $l_{d}$ in the gain $2/l_{d}^{2}$ is that it provides a kind of look-ahead distance. As is customary in the pure pursuit control literature, the look-ahead distance is tuned to be stable at several constant speeds. If the look-ahead distance is a function of the speed of the vehicle, then gain-scheduling and linear parameter varying control (LPV) techniques can be used to analyze the stability of the resulting closed-loop dynamics (see, e.g., the recent work by Kapsalis, et al. (2021)). The nonlinear feedback control law associated with Stanley controller, on the other hand, relies on the cross track error $e_{f_{a}}$ from the center of the front axle to the nearest path point. The intuition behind Stanley control scheme is that the larger the cross-track error from the path, the further the steering of the wheels toward the path. Despite the demonstrated superiority of Stanley controller in the DARPA challenge, in extremely rare maneuvers, pure pursuit controllers demonstrate more robustness with respect to sudden lane changes. On the other hand, pure pursuit controllers have shown miserable failures under paths with fast varying curvatures (see, Snider (2009) for further details).

By studying such extremes of Stanley and pure pursuit control schemes in our simulations, we highlight the results that can be expected for low-level control of military autonomous convoys across the spectrum of trajectory-tracking control schemes. The network topology of the convoy is configured so that each vehicle only has information of its adjacent leader and follower. We compared the convoy controllers within the three categories defined by the framework: goal specification, spacing policy, and system parameters. In goal specification, we are concerned with the amount of deviation of autonomous convoy members from a given specified path. In spacing policy, we are concerned with maintaining a desired distance between the autonomous convoy members. Finally, in system parameters, we are concerned with the controller parameters that need to be tuned to achieve a given control objective. The results of the comparison are as follows.

### Goal Specification

**FIGURE 5:**
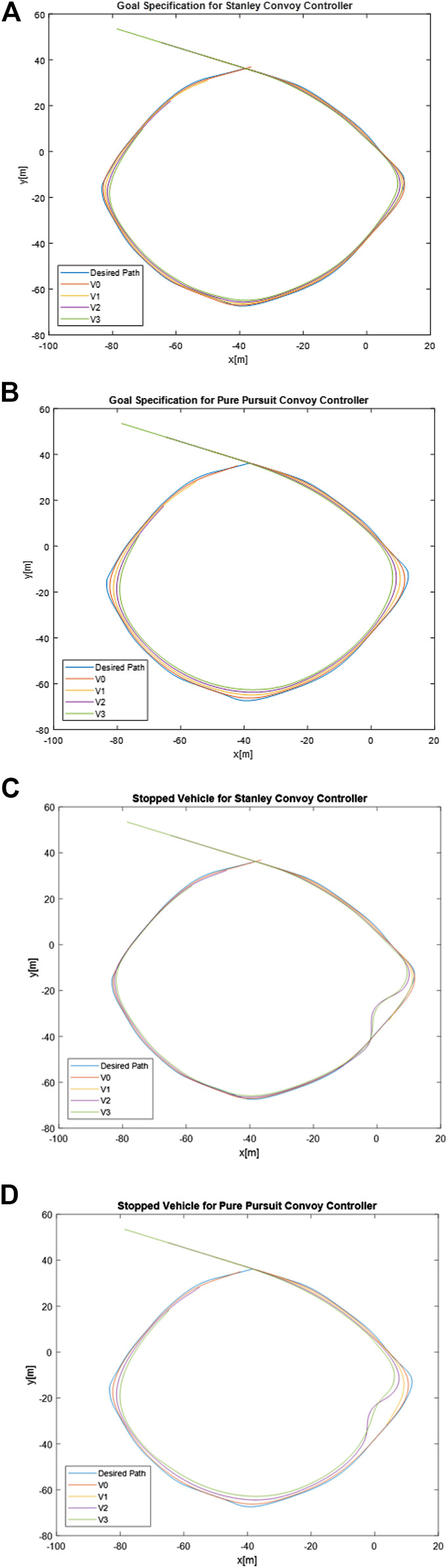
Planned path and taken path of convoy vehicles for Requirement 1 using **(A)** Stanley and **(B)** Pure Pursuit and Requirement 6 using **(C)** Stanley and **(D)** Pure Pursuit.

To compare performance in Goal Specification, a circular route was created. Per the framework detailed in this effort, vehicle offset from the desired path is the most appropriate metric to use for comparison between convoy controllers for the Goal Specification requirements. The two Goal Specification requirements that we will examine in this comparison are Requirement 1 and Requirement 6. The overall convoy vehicle positions for the two different convoy controllers applied to Requirement 1 are shown in Figure 5A and Figure 5B. Circular paths provide proper test cases where one is interested in studying the effectiveness of the proposed controllers in minimizing the deviation of each autonomous convoy member from the desired paths. The Spacing Policy studies, which are concerned with maintaining proper distances in-between the convoy members, are discussed in the next section (i.e., Section 6.2).

**FIGURE 6:**
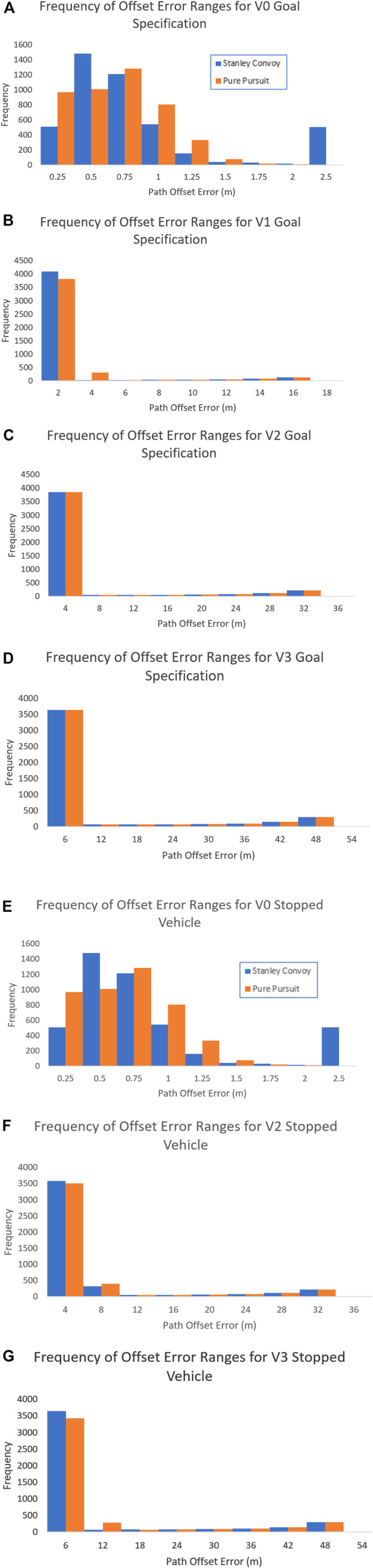
Frequency of offset error ranges for Requirement 1 for **(A)** Vehicle 0, **(B)** Vehicle 1, **(C)** Vehicle 2, and **(D)** Vehicle 3 and Requirement 6 for **(E)** Vehicle 0, **(F)** Vehicle 2, and **(G)** Vehicle 3.

To compare path offset performance of the convoy controllers, we leveraged metrics used in positional tracking (Needham and Boyle, 2003) due to the similarities between vehicle path following and trajectory tracking in computer vision. The metrics and results for Goal Specification are shown in Table 3. In addition, Figure 6A, Figure 6B, Figure 6C, and Figure 6D demonstrate the frequency distribution of the path offset to show the spread of the error for Requirement 1.

**TABLE 3:** Comparison metrics for path offset error.

		Vehicle 0 path offset error (m)		Vehicle 1 path offset error (m)		Vehicle 2 path offset error (m)		Vehicle 3 path offset error (m)	
		Stanley	Pure Pursuit	Stanley	Pure Pursuit	Stanley	Pure Pursuit	Stanley	Pure Pursuit
Req. 1	Mean	0.7149	0.5975	1.7203	2.0791	4.0645	4.5741	7.1026	7.9936
	St. Dev	0.5568	0.3086	3.1583	3.0689	7.9315	7.7624	13.4847	13.1704
Req. 6	Mean	0.7156	0.5725	N/A	N/A	4.2375	4.7396	7.4087	8.2358
	St. Dev	0.5571	0.3085	N/A	N/A	7.9525	7.7913	13.5224	13.1862

As evident in Table 3, the lead convoy vehicle performed better in adhering to the desired path for the Pure Pursuit controller as opposed to the Stanley controller, with a lower mean offset error and standard deviation. However, the subsequent Stanley convoy followers had a lower mean offset error when compared to Pure Pursuit, with similar distributions of error, as shown in Figures 6B–D. This indicates that if the requirement of Goal Specification is the most important factor, the convoy overall performs better utilizing a Stanley convoy controller. While the lead vehicle performed better with Pure Pursuit compared to Stanley, we are comparing autonomous following performance in an AGC and not the performance of solely the lead vehicle.

To compare path offset performance of the convoy controllers for Requirement 6, we utilized the same path and convoy controllers but modified the experiment so that Vehicle 1 stopped motion at 17.71 s into the run. At that time, Vehicle 2 modifies its leader to ignore Vehicle 1 and follow Vehicle 0 directly, while Vehicle 3 continues to follow Vehicle 2 per the initial setup. The overall convoy vehicle positions for the two different convoy controllers applied to Requirement 6 are shown in Figure 5C and Figure 5D. As with the initial Goal Specification experiment, we leveraged metrics used in positional tracking. The metrics and results for Stopped Vehicle are shown in Table 3. In addition, Figure 6E, Figure 6F, and Figure 6G show the frequency distribution of the path offset to show the spread of the error.

For the Stopped Vehicle experiment, the results of Vehicle 1 were omitted due to that vehicle leaving the convoy shortly after the experiment began. As evident in Table 3, the lead convoy vehicle once again performed better in adhering to the desired path for the Pure Pursuit controller as opposed to the Stanley controller, with a lower mean offset error and standard deviation. Likewise, the subsequent Stanley convoy followers performed better in terms of mean offset error when compared to Pure Pursuit, with similar distributions of error, as shown in Figures 6F, G. This indicates that if the requirement of Stopped Vehicle convoy recovery is the most important factor, the convoy overall performs better utilizing a Stanley convoy controller yet again.

Overall, the Goal Specification requirements favored the Stanley convoy controller in terms of performance based on the metrics discussed in this effort.

### Spacing Policy

To compare performance in Spacing Policy, a straight-line path was created in ANVEL. As previously described, a convoy system’s string stability is the most appropriate metric to use for comparison between convoy controllers for Spacing Policy requirements. The Spacing Policy requirement that we will examine in this comparison is Requirement 4. To compare performance of this requirement between the convoy controllers, two gap distances were used: 15 and 30 m. For both the Stanley convoy controller and the Pure Pursuit convoy controller, a test run with a 15 m gap distance was recorded, followed by a run with a 30 m gap distance, both with a convoy speed set at 8 m/s in both instances. Because the requirement is for adjustable gap distance, we will compare how performance changes between the 15 and 30 m for both controllers to determine which one better handled adjusting of distances. Although the spacing policy simulations are being done along a straight-line path for the sake of brevity, the convoy controllers are general enough to regulate the distancing between the autonomous convoy members in more complex situations such as roads on curvy hills. In particular, one can use the longitude and latitude of the autonomous convoy members and then compute their distance from the Haversine equation (Amer et al., 2018).

**FIGURE 7:**
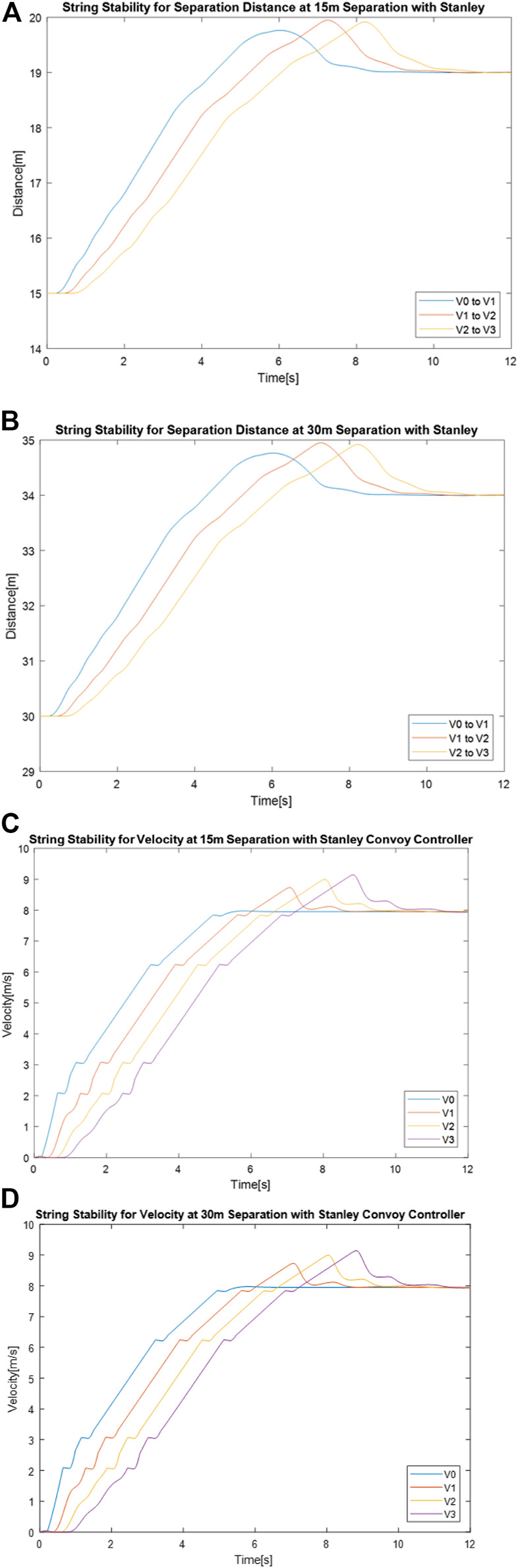
Stanley convoy controller performance for separation distance between vehicles with a gap distance of **(A)** 15 m and **(B)** 30 m and velocity of vehicles over time with a gap distance of **(C)** 15 m and **(D)** 30 m.

Figures 7A, B shows separation distance between vehicles over time for 15 and 30 m using the Stanley convoy controller, while Figure 7C and Figure 7D shows the vehicle velocity over time for those same convoy settings.

As seen in Table 4, the performance characteristics between 15 and 30 m for the Stanley controller did not change. While the minimum and maximum separation distance increased when the convoy gap distance was changed from 15 to 30 m, the overall range of the distances remained consistent. This indicates that adjusting the gap distance had no detrimental effect on performance regarding separation distance error. In addition, the changes in velocity between the 15 m gap setting and the 30 m gap setting were negligibly low, as shown by the difference between the mean values in Table 5. Comparing the performance of the two systems with four significant figures shows no change in velocity for all vehicles, indicating no detrimental effect on velocity performance in regard to adjusting gap distance. Overall, adjusting gap distance showed little to no detrimental effect on convoy performance when using the Stanley convoy controller.

**TABLE 4:** Gap range for different gap settings.

		15 m Gap setting			30 m Gap setting		
		V0 to V1	V1 to V2	V2 to V3	V0 to V1	V1 to V2	V2 to V3
Stanley	Min gap (m)	15.000	15.000	15.000	30.000	30.000	30.000
	Max gap (m)	19.762	19.947	19.917	34.762	34.947	34.917
	Range (m)	4.762	4.947	4.917	4.762	4.947	4.917
Pure Pursuit	Min gap (m)	15.000	15.000	14.999	30.000	30.000	30.000
	Max gap (m)	19.792	19.944	19.892	34.782	34.936	34.919
	Range (m)	4.792	4.944	4.892	4.782	4.936	4.919

**TABLE 5:** Average velocity per vehicle using for different gap settings.

		Vehicle 0	Vehicle 1	Vehicle 2	Vehicle 3
Stanley	Average velocity for 15 m gap (m/s)	7.127	6.927	6.727	6.527
	Average velocity for 30 m gap (m/s)	7.127	6.927	6.727	6.527
	Difference (m/s)	0.000	0.000	0.000	0.000
Pure Pursuit	Average velocity for 15 m gap (m/s)	6.577	6.244	5.91	5.577
	Average velocity for 30 m gap (m/s)	6.577	6.244	5.91	5.577
	Difference (m/s)	0.000	0.000	0.000	0.000

**FIGURE 8:**
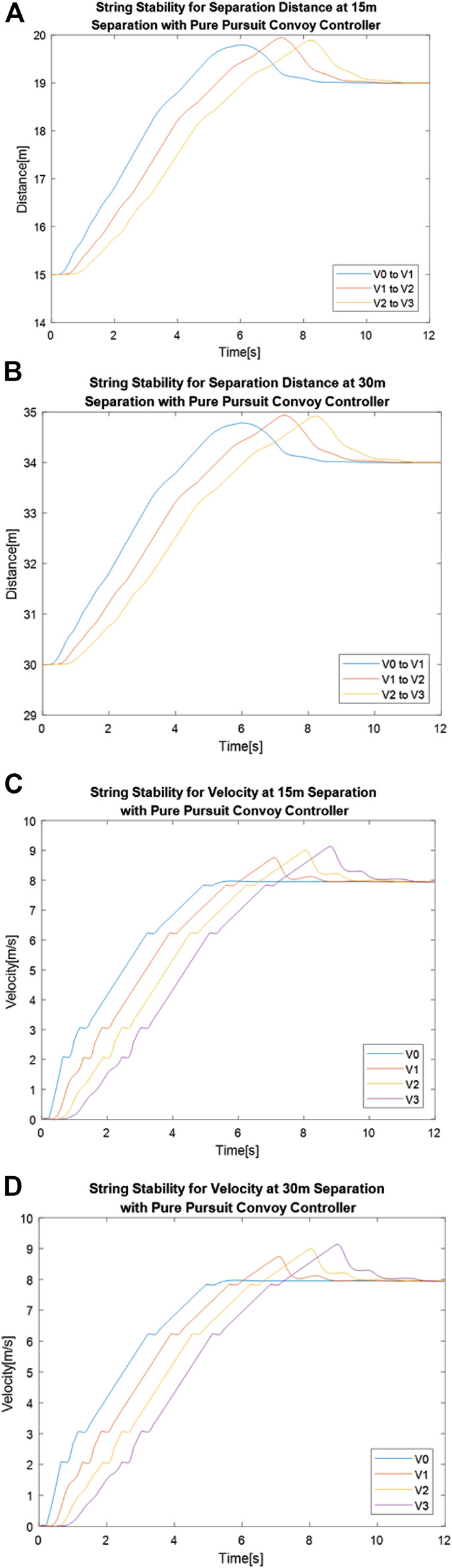
Pure Pursuit convoy controller performance for separation distance between vehicles with a gap distance of **(A)** 15 m and **(B)** 30 m and velocity of vehicles over time with a gap distance of **(C)** 15 m and **(D)** 30 m.

Figures 8A, B shows separation distance between vehicles over time for 15 and 30 m using the Pure Pursuit convoy controller, while Figure 8C and Figure 8D shows the vehicle velocity over time for those same convoy settings.

When using the Pure Pursuit convoy control, there were performance differences between the 15 and 30 m gap distance setting. Unlike in the Stanley convoy controller case, the overall range of the distances between vehicles changes between the two gap distance settings, as seen in Table 4. The range of separation distances between “V1 to V2” and “V2 to V3” increases by 0.35 m when the gap distance is increased from 15 to 30 m, indicating that an increase in gap distance affects how well the system can correct for the propagation of separation distance error. Despite the performance differences in separation distance, the changes in velocity between the 15 m gap setting and the 30 m gap setting were negligibly low, as shown by the difference between the mean values in Table 5. Comparing the performance of the two systems with four significant figures shows no change in velocity for all vehicles, indicating no detrimental effect on velocity performance in regard to adjusting gap distance. Overall, adjusting gap distance showed a slight detrimental effect on convoy performance for string stability of the separation distance when using the Pure Pursuit convoy controller.

When examining both Stanley and Pure Pursuit performance as a whole given spacing policy requirements, the Stanley convoy controller performs better when considering the metrics discussed in this effort.

### System Parameters

To compare performance in System Parameters, the same straight-line path used in Spacing Policy was used for test runs. As previously described, a convoy system’s string stability is the most appropriate metric to use for comparison between convoy controllers for System Parameter requirements. The System Parameter requirement that we will examine in this comparison is Requirement 3. To compare performance of this requirement between the convoy controllers, two velocities were used: 10 and 20 m/s. For both the Stanley convoy controller and Pure Pursuit convoy controller, an experiment was run with a desired convoy velocity of 10 m/s, followed by a run with a desired convoy velocity of 20 m/s. Both runs set the separation distance at 15 m. Because the requirement is for adjustable rate of march, we will compare how performance changes between 10 and 20 m/s for both controllers to determine which one better handled adjusting of rates of march.

**FIGURE 9:**
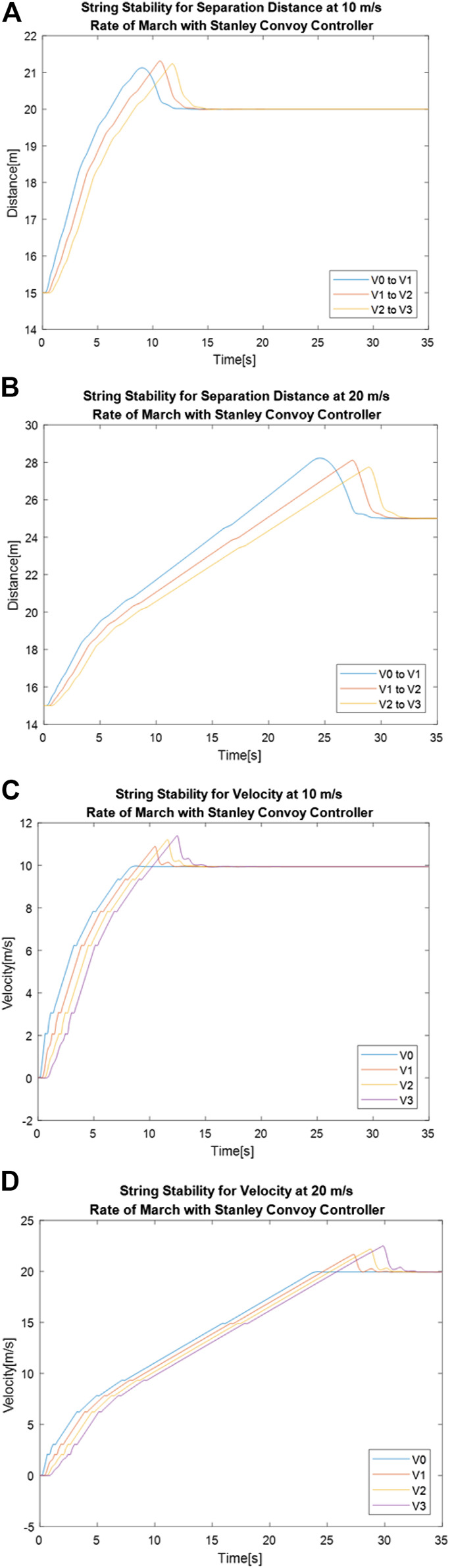
Stanley convoy controller performance for separation distance between vehicles with a rate of march of **(A)** 10 m/s and **(B)** 20 m/s and velocity of vehicles over time with a gap distance of **(C)** 10 m/s and **(D)** 20 m/s.

Figures 9A, B shows separation distance between vehicles over time for 10 and 20 m/s rate of marches respectively, using the Stanley convoy controller, while Figure 9C and Figure 9D shows the vehicle velocity over time for those same convoy settings. The convoy achieves string stability when the rate of march is increased from 10 m/s, as seen in Figure 9A, to 20 m/s, as seen in Figure 9B, for the Stanley convoy controller. This can be seen by looking at the peaks of the separation distance measurements and noting that the error decreases throughout the convoy followers, as seen in Figure 9B, as opposed to increasing, as seen in Figure 9A. This is noted in Table 6 by reviewing the percentage changes of maximum gap distances. As seen in Figure 9C and Figure 9D, the Stanley convoy controller fails to achieve string stability for velocity regardless of the rate of march. Table 7 shows the maximum velocities member vehicles reached, along with the percentage difference between the maximum velocities between vehicles. While both rates of march failed to achieve string stability for velocity, the percentage changes show that the 10 m/s Rate of March created greater error propagation down the line of the convoy.

**TABLE 6:** Maximum gap distance for different Rate of March settings.

		10 m/s rate of March			20 m/s rate of March		
		V0 to V1	V1 to V2	V2 to V3	V0 to V1	V1 to V2	V2 to V2
Stanley	Maximum gap (m)	21.125	21.312	21.235	28.226	28.107	27.741
	% Change from previous gap distance	N/A	0.876%	−0.361%	N/A	−0.424%	−1.319%
Pure Pursuit	Maximum gap (m)	21.166	21.302	21.202	28.321	28.062	27.672
	% Change from previous gap distance	N/A	0.64%	−0.47%	N/A	−0.92%	−1.41%

**TABLE 7:** Maximum vehicle velocities for different Rate of March settings.

			Vehicle 0	Vehicle 1	Vehicle 2	Vehicle 3
Stanley	10 m/s Rate of March	Maximum velocity (m/s)	9.968	10.887	11.212	11.390
		% Change from max velocity of preceding vehicle	N/A	8.441%	2.898%	1.562%
	20 m/s Rate of March	Maximum velocity (m/s)	19.968	21.680	22.194	22.494
		% Change from max velocity of preceding vehicle	N/A	7.896%	2.314%	1.337%
Pure Pursuit	10 m/s Rate of March	Maximum velocity (m/s)	9.968	10.908	11.218	11.382
		% Change from max velocity of preceding vehicle	N/A	8.62%	2.76%	1.45%
	20 m/s Rate of March	Maximum velocity (m/s)	19.968	21.709	22.198	22.488
		% Change from max velocity of preceding vehicle	N/A	8.02%	2.21%	1.29%

**FIGURE 10:**
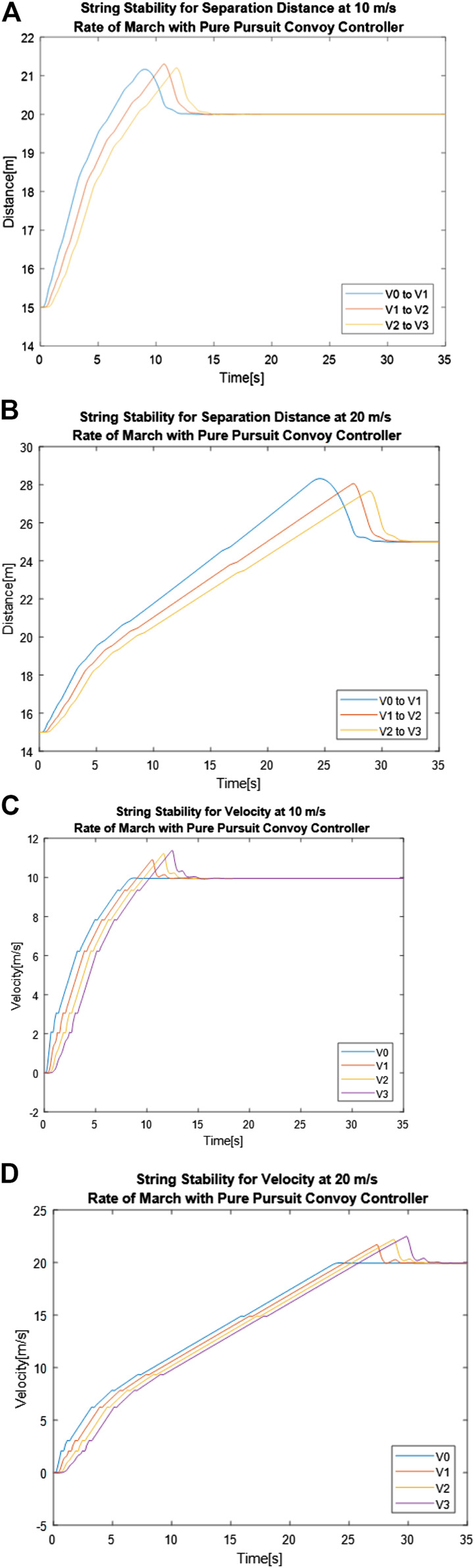
Pure Pursuit convoy controller performance for separation distance between vehicles with a rate of march of **(A)** 10 m/s and **(B)** 20 m/s and velocity of vehicles over time with a gap distance of **(C)** 10 m/s and **(D)** 20 m/s.

Figures 10A, B shows separation distance between vehicles over time for 10 and 20 m/s rates of marches respectively using the Pure Pursuit convoy controller, while Figure 10C and Figure 10D shows the vehicle velocity over time for those same convoy settings. These results tracked closely to what was seen with the Stanley convoy controller. The convoy achieves string stability when the rate of march is increased from 10 to 20 m/s for the Pure Pursuit convoy controller, much like with the Stanley convoy controller. This can be seen once again by looking at the peaks of the separation distance measurements and noting that the error decreases throughout the convoy followers, as seen in Figure 10A and Figure 10B. This is noted in Table 6 by reviewing the percentage changes of maximum gap distances. While the 10 m/s rate of march shows a positive and negative fluctuation of the separation distance, the 20 m/s rate of march only decreases, indicating that error does not propagate through. As seen in Figure 10C and Figure 10D, the Pure Pursuit convoy controller fails to achieve string stability for velocity regardless of the rate of march. Table 7 shows the maximum velocities member vehicles reached, along with the percentage difference between the maximum velocities when comparing vehicles with their predecessor. While both rates of march failed to achieve string stability for velocity, the percentage changes show that the 10 m/s Rate of March created greater error propagation down the line of the convoy.

Overall, adjusting the rate of march affected the convoy controllers differently depending on the metric being analyzed. From the perspective of separation distance, changing the rate of march from 10 to 20 m/s reduced the propagation of error down the convoy in both Stanley and Pure Pursuit, but the reduction of error was greater for Pure Pursuit. This indicates that Pure Pursuit performs better in increasing the rate of march from the perspective of maintaining the desired separation difference. From the perspective of velocity, both the Stanley and Pure Pursuit convoy controller showed string instability, regardless of the rate of march. Neither controller showed a consistent reduction of instability between vehicles in adjusting the rate of march, indicating that in this application, separation distance should be the determining factor of performance. This means that for the System Parameters requirement analyzed here, the Pure Pursuit convoy controller should be used for optimal performance.

## Discussion

By reviewing and analyzing both Army doctrine and the field of AGC research, we were able to develop a framework for comparison of AGC performance as it relates to military convoy needs. With humanitarian military efforts relying on convoys for supply delivery, medical practitioner transportation, and medical evacuation, it is important to understand what autonomous convoy technology best serves the needs of healthcare resource delivery. Requirements 1, 2, and 6 directly relate to Goal Specification objectives of autonomous convoy control framework. Furthermore, Requirements 4 and 5 are related to Spacing Policy objective of the autonomous convoy control framework. As it can be seen from the simulation results presented, Stanley Controller demonstrates a more robust performance in fulfilling the Spacing Policy requirement over a range of gap distances. Finally, Requirements 3 and 7 are related to System Parameters. As it is demonstrated in simulation, the Pure Pursuit Controller had a better performance in fulfilling the Spacing Policy requirement.

Even with the framework however, comparative performance is highly dependent on the requirement needs to be prioritized. No sole factor singularly defines the quality of a convoy, and considerations such as terrain, hostile forces, and size of the convoy elements may change what can be considered the best choice for an AGC solution working toward military requirements. The purpose of the framework is to provide a way to compare different AGC efforts, but the user must have a strong understanding of the baseline mission needs to make a meaningful comparison. For a healthcare delivery perspective, a convoy commander will have to make the same considerations, with added logistical complexity of the placement of medical vehicles within the convoy. The intended outcome of this effort is to better understand how AGC technologies perform relative to one another given the needs of healthcare delivery in a medical context, in order to have metrics to improve upon between the research and development of new systems.

## Conclusion

In this effort, we performed a review of Army doctrine to derive requirements for convoy performance toward delivery of healthcare resources. After discussing a hierarchical decision-making, we argued for using nonlinear battlefield techniques for delivering healthcare logistics to remote pandemic outbreak areas. Through examining publicly available doctrine, we identified seven key requirements to be met when in developing AGCs for a military context. By doing a survey of AGC efforts, we found that metrics related to spacing policy and string stability were commonly used and could be leveraged as the basis for a framework of performance comparison between different AGC systems. With that framework in hand, we showed a sample application, comparing the performance of a Stanley convoy controller and a Pure Pursuit convoy controller. By creating this framework, we look to enable future AGC development efforts to properly baseline and compare performance between existing systems, to find optimal solutions for delivery of healthcare resources using AGCs.

## Footnotes

1 In this article, we use the preferred spelling of “Soldier” under the U.S. DoD guidance

2 https://www.michigan.gov/egle/0,9429,7-135-3313_71618-545670--,00.html

3 See the following DoD News network article for more details: https://www.defense.gov/Explore/News/Article/Article/2393298/military-to-play-logistics-only-role-in-covid-19-vaccine-effort/

4 In the context of the control of autonomous vehicle platoons, this is known as an autonomous rendevousz requirement

5 Numerous adaptive control techniques such as extremum seeking control (Dadras, 2017) and model reference adaptive control (MRAC) (Dixit et al., 2020; Montanaro et al., 2020; Dianati et al., 2020; Mouzakitis et al., 2020; Fallah et al., 2020) can be employed to achieve this requirement in the context of AGCs

6 We remark that the terms “measures”/“metrics” are used interchangeably in this article.
